# Early Life Stress-Related Elevations in Reaction Time Variability Are Associated with Brain Volume Reductions in HIV+ Adults

**DOI:** 10.3389/fnbeh.2018.00006

**Published:** 2018-01-30

**Authors:** Uraina S. Clark, Miguel Arce Rentería, Rachal R. Hegde, Susan Morgello

**Affiliations:** ^1^Department of Neurology, Icahn School of Medicine at Mount Sinai, New York, NY, United States; ^2^Department of Psychology, Fordham University, New York, NY, United States

**Keywords:** reaction time variability, adverse childhood experiences, childhood abuse, childhood trauma, gray matter, white matter, subjective cognitive complaints

## Abstract

There is burgeoning evidence that, among HIV+ adults, exposure to high levels of early life stress (ELS) is associated with increased cognitive impairment as well as brain volume abnormalities and elevated neuropsychiatric symptoms. Currently, we have a limited understanding of the degree to which cognitive difficulties observed in HIV+ High-ELS samples reflect underlying neural abnormalities rather than increases in neuropsychiatric symptoms. Here, we utilized a behavioral marker of cognitive function, reaction time intra-individual variability (RT-IIV), which is sensitive to both brain volume reductions and neuropsychiatric symptoms, to elucidate the unique contributions of brain volume abnormalities and neuropsychiatric symptoms to cognitive difficulties in HIV+ High-ELS adults. We assessed the relation of RT-IIV to neuropsychiatric symptom levels and total gray and white matter volumes in 44 HIV+ adults (26 with high ELS). RT-IIV was examined during a working memory task. Self-report measures assessed current neuropsychiatric symptoms (depression, stress, post-traumatic stress disorder). Magnetic resonance imaging was used to quantify total gray and white matter volumes. Compared to Low-ELS participants, High-ELS participants exhibited elevated RT-IIV, elevated neuropsychiatric symptoms, and reduced gray and white matter volumes. Across the entire sample, RT-IIV was significantly associated with gray and white matter volumes, whereas significant associations with neuropsychiatric symptoms were not observed. In the High-ELS group, despite the presence of elevated neuropsychiatric symptom levels, brain volume reductions explained more than 13% of the variance in RT-IIV, whereas neuropsychiatric symptoms explained less than 1%. Collectively, these data provide evidence that, in HIV+ High-ELS adults, ELS-related cognitive difficulties (as indexed by RT-IIV) exhibit strong associations with global brain volumes, whereas ELS-related elevations in neuropsychiatric symptoms appear to contribute minimally to these cognitive difficulties. Such findings support a growing body of evidence indicating that high ELS exposure is a significant risk factor for neurocognitive dysfunction in HIV+ adults. Further, these data highlight the need to better understand how ELS-related pathophysiological mechanisms contribute to volumetric and other neural abnormalities in HIV+ individuals.

## Introduction

Recent studies indicate that exposure to high levels of early life stress (ELS) can increase the risk of cognitive impairment among HIV+ adults (Clark et al., 2012; Spies et al., 2012; Womersley et al., 2017). Difficulties in the areas of executive function and processing speed have been observed in HIV+ adults with high ELS (Clark et al., 2012; Spies et al., 2012, 2016) and have been linked to abnormalities in brain volume (Clark et al., 2012; Spies et al., 2016). Yet, few studies have been conducted in this area, resulting in an incomplete understanding of the cognitive difficulties that are associated with high ELS exposure in HIV+ adults. One unresolved question is the degree to which cognitive difficulties in HIV+ High-ELS adults reflect underlying neural abnormalities as opposed to neuropsychiatric symptoms (e.g., depression, post-traumatic stress disorder [PTSD]), which have detrimental effects on cognitive functions (Ode et al., 2011; Swick et al., 2012; Fellows et al., 2013). This is particularly concerning, given recent findings indicating that high ELS exposure is associated with elevated neuropsychiatric symptoms in HIV+ adults (Clark et al., 2017).

There is thus a need to determine the unique contributions of ELS-related neuropsychiatric symptoms and neural abnormalities to cognitive difficulties in HIV+ adults. Considering the evidence that neuropsychiatric symptoms are a prominent component of the prodromal phases of Alzheimer’s disease and other dementias (Lyketsos et al., 2011; Gallagher et al., 2017), gaining greater clarity on these issues may help to further delineate underlying factors associated with HIV-associated neurocognitive disorders. Although neuropsychiatric symptoms have known associations with cognitive dysfunction, to date, only two prior studies have examined the contribution of neuropsychiatric symptoms to cognitive difficulties in HIV+ High-ELS adults (Clark et al., 2012; Spies et al., 2012). Results from these studies suggest minimal effects of depression levels on cognitive difficulties in HIV+ High-ELS adults; however, the effects of additional neuropsychiatric symptoms commonly elevated in High-ELS samples, such as PTSD-related symptoms and current stress, have not been systematically assessed. While the aforementioned studies utilized neuropsychological batteries that were tailored to detect HIV-related cognitive decline, the current study takes a novel approach by utilizing a sensitive behavioral measure of cognitive function, reaction time intra-individual variability (RT-IIV), to examine these issues in greater depth.

Reaction time intra-individual variability is a measure of an individual’s variability in response times summarized across a number of trials in a task. Elevations in RT-IIV arise, in part, due to an increase in attentional lapses (Leth-Steensen et al., 2000; Hervey et al., 2006; Tamm et al., 2012), which are thought to reflect an inefficient regulation of cognitive processes (West et al., 2002; Chuah et al., 2006; Ode et al., 2011). RT-IIV is thus considered a marker of cognitive instability (Fjell et al., 2011). Accordingly, RT-IIV provides information about cognitive processes that is distinct from that captured by traditional neuropsychological measures. There is some indication that RT-IIV may even be more sensitive to subtle cognitive impairments than standardized neuropsychological measures (Collins and Long, 1996). Notably, prior studies have used RT-IIV measures to differentiate between patient groups with depression, trauma, and other neuropsychiatric disorders (Kaiser et al., 2008; Swick et al., 2012). RT-IIV measures have also been used to differentiate between individuals with and without neurological conditions (e.g., traumatic brain injury, mild cognitive impairment) (Collins and Long, 1996; MacDonald et al., 2006; Dixon et al., 2007; Duchek et al., 2009). Moreover, RT-IIV has been identified as a valuable indicator of compromised neural integrity (MacDonald et al., 2006; Nilsson et al., 2014). RT-IIV is sensitive to global white matter volume reductions (Walhovd and Fjell, 2007; Jackson et al., 2012), as well as frontal-lobe abnormalities (e.g., white matter hyperintensities) (Bunce et al., 2007, 2010; MacDonald et al., 2012; Lovden et al., 2013).

Only three prior studies have investigated RT-IIV in HIV+ samples (Levine et al., 2006, 2008; Ettenhofer et al., 2010). These studies indicate that elevated RT-IIV is associated with greater cognitive impairment and poorer clinical outcomes (reduced antiretroviral adherence, greater immunological dysfunction) (Levine et al., 2008; Ettenhofer et al., 2010). These associations do not appear to be a function of slowed RTs, which are common in HIV+ samples, as RT-IIV exhibits independent associations with cognitive functions, even when controlling for RT latency in HIV+ adults (Ettenhofer et al., 2010).

There is thus strong evidence indicating that RT-IIV is sensitive to the presence of neuropsychiatric symptoms (e.g., stress exposure, depression), as well as changes in cognitive function and neural integrity (e.g., brain volume). Accordingly, this behavioral measure is well suited to the current investigation, as the primary aim of this study is to examine whether ELS-related cognitive reductions observed in HIV+ High-ELS adults are more strongly associated with global brain volumes than with neuropsychiatric symptoms. Building on prior findings (Clark et al., 2012, 2017; Spies et al., 2012, 2016), we hypothesized that HIV+ adults with high ELS, relative to those with low ELS, would demonstrate elevations in RT-IIV, greater neuropsychiatric symptom levels, and lower total gray and white matter volumes. We further hypothesized that elevations in RT-IIV would be more strongly associated with brain volume than with neuropsychiatric symptom levels. As a test of the functional validity of our RT-IIV measure, we examined the relation between RT-IIV and a self-report measure of cognitive function. Based on prior findings (Ettenhofer et al., 2010), we expected to observe strong correlations between RT-IIV and subjective ratings of cognitive function. Our final goal was exploratory in nature and assessed potential contributory factors associated with brain volume abnormalities in this HIV+ sample. Consistent with our general hypothesis, we predicted that degree of ELS exposure, more so than neuropsychiatric symptom levels, would be associated with reductions in brain volume. We further predicted that a measure of historical HIV-disease severity – nadir CD4 levels (i.e., the lowest ever T-cell count on record) – would also demonstrate independent associations with brain volume (Clark and Cohen, 2010; Cohen et al., 2010; Jernigan et al., 2011; Clark et al., 2012, 2015; Guha et al., 2016). Collectively, such analyses aim to further clarify our understanding of the neurocognitive correlates of high ELS exposure in HIV+ adults.

## Materials and Methods

### Participants

We recruited 44 HIV+ adults from the Icahn School of Medicine at Mount Sinai in New York, NY, United States and The Miriam Hospital in Providence, RI, United States. One investigator (USC) oversaw all procedures. The Institutional Review Boards at the Icahn School of Medicine at Mount Sinai and The Miriam Hospital approved this research. All participants gave their informed written consent and were financially compensated for their time.

Inclusion criteria were HIV-positive status, right-handedness, completion of 8 or more years of education, and being a native English speaker. HIV serostatus was documented by ELISA and confirmed by Western blot test. All participants obtained a score of ≥24 points on the Mini-Mental State Exam (MMSE) (Folstein et al., 1975). Exclusion criteria included reported history of uncorrected abnormal vision; developmental disability; learning disability; major psychiatric illness (e.g., bipolar disorder, posttraumatic stress disorder); neurological illness affecting the central nervous system (e.g., stroke, progressive multifocal leukoencephalopathy); and traumatic head injury with loss of consciousness >10 min. Substance use exclusion criteria were reported current alcohol dependence; use of heroin/opiates or any intravenous drug within the past 6 months; use of cocaine within the past month; and positive urine toxicology at the time of assessment (cocaine, opiates, methamphetamine, amphetamine, benzodiazepine, barbiturates, methadone, oxycodone).

### Demographic Measures

HIV disease duration, nadir CD4 levels (i.e., the lowest ever CD4 T-cell count), and antiretroviral (ARV) use were obtained via self-report and verified against the medical record. Current CD4 levels and plasma HIV viral loads (HIVL) were obtained from medical records. All participants were prescribed ARV medications. Participants were assessed for hepatitis C virus (HCV) infection, defined as positive HCV antibody. Alcohol and drug use histories for all participants were quantified using the Kreek-McHugh-Schluger-Kellogg scale (KMSK) (Kellogg et al., 2003), which provided three subscales characterizing lifetime consumption of alcohol (KMSK-A), cocaine (KMSK-C), and opiates (KMSK-O). The Wechsler Test of Adult Reading (WTAR) estimated premorbid levels of intellectual function (Wechsler, 2001); scaled scores were derived using published normative data. See **Table 1** for group characteristics.

**Table 1:** Demographic, neuropsychiatric, and cognitive characteristics of the participant groups.

	HIV+ Low-ELS (*N* = 18)		HIV+ High-ELS (*N* = 26)					
	*Mean*	*SD*	*Mean*	*SD*	*F/t/U/χ^2^*	*df*	*p*	*ES*
**Demographic characteristics**								
Recruitment Site (% New York, NY, United States)	67		65		0.01	1	0.930	0.013^b^
% Providence, RI, United States	33		35					
Age (years)	44.44	9.42	46.08	9.36	0.57	42	0.574	0.008
Education (years)	13.08	2.68	13.73	2.51	0.82	42	0.417	0.015
% Male	50		65		1.04	1	0.307	0.154^b^
Mini-Mental State Exam (/30)	29.06	1.00	28.38	1.53	1.76	41.91	0.085	0.071
WTAR (SS)	99.89	14.34	96.58	19.01	0.63	42	0.535	0.010
Racial composition (% Caucasian)	17		27				0.489	0.120^b^
% African American	78		65					
% Asian American			4					
% Native American			4					
% Bi/Multiracial	6							
Ethnic composition (% Hispanic)	22		12				0.419	0.144^b^
% Hepatitis C positive	17		12				0.683	0.066^b^
KMSK – Alcohol (/13)	7.78	4.11	7.08	3.38	0.62	42	0.539	0.008
KMSK – Cocaine (/16)	5.39	7.16	7.88	6.37	1.21	42	0.231	0.032
KMSK – Opiate (/13)	1.67	4.02	0.62	1.68	1.20	42	0.238	0.025
% With positive marijuana toxicology	22		28				0.736	0.065^b^
Number of ACEs	1.17	0.86	5.23	2.27	8.32	34.24	<0.001	0.622
**HIV-disease measures**								
Nadir CD4 (cells/μl)	261.59	262.97	210.58	214.76	202.50		0.646	0.005
Current CD4 (cells/μl)	594.28	298.40	608.85	272.19	0.17	42	0.868	0.001
Current log_10_ HIVL	2.21	1.23	1.75	0.87	165.50		0.161	0.047
% with HIVL below 50 copies/ml	53		65		0.67	1	0.415	0.124^b^
Length of HIV infection (years)	14.56	6.64	16.00	7.22	0.67	42	0.504	0.011
% on ARV medications	100		100					
**Neuropsychiatric measures**								
Depression – CESD (/60)	6.61	6.18	14.62	11.51	2.98	39.96	0.005	0.175
Current stress – PSS (/56)	14.11	5.70	19.35	8.38	2.30	42	0.026	0.127
PTSD symptoms – PCLC (/85)	24.33	10.02	33.19	12.72	2.47	42	0.018	0.137
Neuropsychiatric composite (*z*-score)	–0.43	0.60	0.29	0.98	3.02	41.54	0.004	0.178
**Cognitive measures**								
1-back trial response rate (%)	96.86	4.37	97.29	4.36	0.31	39	0.758	0.002
1-back A′ (signal detection)	0.92	0.08	0.90	0.08	151.00		0.161	0.049
1-back mean RT latency (ms)^a^	781.44	134.21	817.11	135.45	1.00	1,38	0.323	0.026
1-back RT-IIV (CoV)^a^	0.23	0.05	0.28	0.07	5.82	1,38	0.021	0.133
MOS-HIV cognitive function (*z*-score)^a^	0.22	0.81	0.13	0.69	0.01	1,26	0.911	0.000

*ELS, early life stress; WTAR, Wechsler Test of Adult Reading; SS, Standard Score; KMSK, Kreek-McHugh-Schluger-Kellogg scale; ACE, adverse childhood events; HIVL, HIV viral load; ARV, antiretroviral; CESD, Center for Epidemiologic Studies Depression Scale; PSS, Perceived Stress Scale; PCLC, PTSD Checklist - Civilian; RT-IIV, reaction time intra-individual variability; CoV, coefficient of variation, a measure of variability where higher values indicate greater variability; MOS-HIV, The Medical Outcomes Study HIV Health Survey; ES, effect size. ^*a*^Analysis includes neuropsychiatric composite index (depression, current stress, PTSD) as a covariate. Measures of effect size (ES) include eta-squared (η^*2*^), partial eta-squared (η_*p*_^*2*^), and the phi (ϕ) coefficient (Cohen, 1988; Lakens, 2013; Corder and Foreman, 2014). For η^*2*^ and η_*p*_^*2*^, values of 0.01, 0.06, and 0.14 indicate small, medium, and large effects, respectively. ^*b*^For ϕ, values of 0.10, 0.30, and 0.50 indicate small, medium, and large effects, respectively.*

### Early Life Stress Quantification

Early life stress exposure was quantified using the Early Life Stress Questionnaire (ELSQ) (Cohen et al., 2006b), which assessed the occurrence of 17 adverse childhood events (ACE) (e.g., physical abuse, sexual abuse, neglect, family conflict, bullying) prior to age 18 years. Low ELS was defined by endorsement of fewer than 3 ACEs, and high ELS was defined as an endorsement of 3 or more ACEs, consistent with prior studies (Cohen et al., 2006a; Paul et al., 2008; Seckfort et al., 2008; Clark et al., 2012, 2017). Using these criteria, 18 participants were classified as having low ELS and 26 were classified as having high ELS. The proportion of Low-ELS and High-ELS participants was similar across testing sites (New York, NY, United States: Low-ELS = 12 [41%], High-ELS = 17 [59%]; Providence, RI, United States: Low-ELS = 6 [40%], High-ELS = 9 [60%]).

### Neuropsychiatric Measures

We examined levels of neuropsychiatric symptoms known to be elevated in HIV+ High-ELS individuals (Clark et al., 2012, 2017) and associated with RT-IIV performance (Kaiser et al., 2008; Ode et al., 2011; Swick et al., 2012). Levels of current depression, stress, and PTSD symptoms were quantified using the Center for Epidemiological Studies-Depression Scale (CESD) (Radloff, 1977), Perceived Stress Scale (PSS) (Cohen et al., 1983), and Posttraumatic Checklist – Civilian (PCLC) (Weathers et al., 1993), respectively. For each participant, z-scores were calculated for each measure based on the mean of the entire sample for that measure; the three z-scores were then averaged to create a composite index score for each participant. The index thus represents the overall degree of neuropsychiatric difficulty reported across all domains assessed. To verify this, we conducted a principal components analysis on the three neuropsychiatric measures (Bartlett’s test: χ^2^= 84.5, *p* < 0.001; KMO = 0.704). Only one component had an eigenvalue over 1; this component explained 83% of the total variance (all communalities >0.73). Across the sample, component scores correlated strongly with composite index scores (*r*[44] = 0.9999, *p* < 0.001) (Supplemental Figure S1), supporting the use of the composite index in subsequent analyses. Higher index scores indicate greater global neuropsychiatric difficulty.

### Reaction Time Task

**FIGURE 1:**
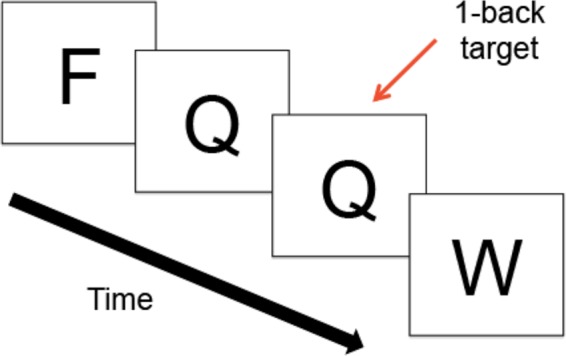
Schematic of the 1-Back Task. During the N-back, a series of consonants is presented on the computer screen one at a time; trials were presented at the rate of 1 every 2 s. Participants indicate whether the letter on the screen is the same as, or different from, the letter presented N-back, where “N” is a specific number of letters.

A computerized N-back paradigm was used to assess mean RT latency and RT-IIV. The N-back is a working memory task in which a series of consonants is presented on the computer screen. Participants indicate whether the letter is the same as, or different from the letter presented N-back, where “N” is a specific number of letters. Our paradigm included several N-back conditions (0-back, 1-back, 2-back, 3-back), where larger Ns provide increased difficulty (greater working memory load). In this study, we examined performance during the 1-back condition (**Figure 1**), based on prior evidence that HIV+ adults, relative to adults without HIV, demonstrate greater RT latencies during the 1-back, without significant reductions in accuracy (Chang et al., 2001). Such findings suggest that the 1-back is sensitive to HIV-related changes in RT without placing excessive demands on cognitive processes. The 1-back thus permits examination of RT abnormalities that arise under conditions that minimize confounding influences of high cognitive demands, thereby better isolating RT performance.

The N-back was completed in the magnetic resonance imaging (MRI) scanner as part of a broader study on brain response in HIV+ adults involving functional MRI. Because the focus of the current study was on volumetric MRI abnormalities, functional data were not included in the current analyses. The N-back paradigm was administered using E-Prime^1^, which recorded trial responses and RTs. Responses were given using a button box placed in the right hand. A total of 64, 1-back trials were administered across four 1-back blocks, which lasted 32 s each and alternated with additional N-back blocks. Trials were presented at the rate of 1 every 2 s (stimuli presentation = 1”; interstimulus interval = 1”). All participants practiced the N-back prior to completing the in-scanner experiment to avoid confounding influence of learning effects on performance. During the in-scanner experiment, accuracy for one participant (High-ELS) was below chance, and two participants (1 Low-ELS; 1 High-ELS) responded to less than 80% of 1-back trials; these three participants were excluded from all analyses involving RT measures. In the remaining sample, correlations between 1-back performance during the brief out-of-scanner practice session and the longer in-scanner task were high (% correct: *r* = 0.52, *p* < 0.001; mean RT: *r* = 0.63, *p* < 0.001; RT-IIV: *r* = 0.33, *p* = 0.037), supporting an examination of in-scanner performance.

Mean RT latency was calculated for each participant across all correct 1-back trials. To measure RT-IIV, we calculated the coefficient of variation (CoV) for each participant using the following formula: CoV = standard deviation across all correct 1-back trials/mean RT (Stuss et al., 2003). CoV thus provides a measure of RT variability that controls for mean RT, where higher scores indicate greater variability. To assess overall performance on 1-back trials, we calculated a nonparametric measure of signal detection, A′ (Snodgrass and Corwin, 1988), which takes into account both omission and commission errors. A′ ranges from 0 to 1, where scores above 0.5 indicate that performance was above chance.

### Structural Brain MRI Acquisition and Analysis

Magnetic resonance imaging scans were conducted at two locations (New York, NY, United States; Providence, RI, United States), each using a Siemens 3T scanner. Whole-brain high-resolution T1-weighted MPRAGE images were acquired in the sagittal plane in all participants (New York, NY, United States: Siemens MAGNETOM Skyra, TE/TR = 2.07/2400 ms, 0.8 mm^3^, FOV = 256 mm × 256 mm; Providence, RI, United States: Siemens TIM TRIO, TE/TR = 2.98/1900 ms, 1 mm^3^, FOV = 256 mm × 256 mm). Gray and white matter volumetric segmentation was performed in an automated manner using the FreeSurfer image analysis suite (v5.3.0) (Fischl et al., 2002). Total gray and white matter volumes (including cerebral and cerebellar regions) were thus derived for each participant. In one participant (Low-ELS), the automated pipeline was unable to run to completion. All other segmentations were visually inspected for accuracy (e.g., segmentation alignment); none were omitted.

Total gray and white matter volumes were adjusted for differences in total intracranial volume (ICV) as follows: Adjusted volume = raw volume – [b × (ICV - mean ICV)], where b is the slope of the regression of the raw volume on ICV. This covariance approach to correcting for ICV has been used frequently in previous studies (Mathalon et al., 1993; Buckner et al., 2004; Raz et al., 2005, 2008; Jackson et al., 2012; Pintzka et al., 2015). Although FreeSurfer provides reliable estimates of brain volumes across scanners (Han et al., 2006; Fennema-Notestine et al., 2007; Han and Fischl, 2007; Dickerson et al., 2008), and multisite volumetric data aggregation has been used successfully in previous studies (Fennema-Notestine et al., 2007; Desikan et al., 2009; Dewey et al., 2010), there is some evidence that differences in MRI acquisition can systematically affect volumetric estimates (e.g., Reig et al., 2009). Accordingly, we adopted a conservative approach and included scanner type as a covariate in all statistical analyses involving volumetric data. As noted above, the proportion of Low-ELS and High-ELS participants was similar across testing sites (**Table 1**).

### Self-Reported Cognitive Function

The HIV Medical Outcomes Survey (MOS-HIV) (Wu, 1996) was administered to assess self-reported rates of cognitive function. The 35-item MOS-HIV is a widely used and accepted measure of health-related quality of life in HIV+ adults (Wu et al., 1997). Four MOS-HIV items contribute to the cognitive function subscale, which assesses the degree to which participants have experienced difficulty concentrating, reasoning, or remembering within the past 4 weeks. Raw subscale scores were transformed to *z*-scores based on published normative data (Wu, 1996). Lower scores indicate greater subjective cognitive difficulty. Due to a slight variation in study protocols across sites, MOS-HIV data were only available from the New York cohort (*N* = 29).

### Statistical Analyses

Differences in demographic variables and neuropsychiatric symptoms between the ELS groups were assessed using independent-samples *t*-tests, chi-square, and Fisher’s exact tests. Mann–Whitney *U* tests were used to compare groups on variables that were not normally distributed (nadir CD4 levels, log_10_ HIVL, 1-back A′). Our main goals were threefold. First, we tested whether the High-ELS group demonstrated greater RT-IIV than the Low-ELS group, using analyses of covariance (ANCOVA) to control for group differences in neuropsychiatric symptoms (composite scores). Partial eta-squared ($\eta_{p}^{2}$) was used as an indicator of effect size, where values of 0.01, 0.06, and 0.14 indicate small, medium, and large effects, respectively (Cohen, 1988). Second, we used a similar approach (ANCOVA) to test for possible group differences in total gray and white matter volumes, while controlling for neuropsychiatric symptoms (composite scores); scanner type was included in these analyses as a covariate, as our data suggested that volume estimates derived from the NY protocol tended to be smaller than those derived from the RI protocol. Third, we examined the extent to which neuropsychiatric symptoms and brain volumes accounted for variance in RT-IIV across the entire sample using hierarchical regression. In each model, RT-IIV was entered as the dependent variable; neuropsychiatric composite scores were entered as the independent variable in the first step, scanner type was entered as a covariate in the second step, and the MRI volume of interest was entered in the third step. Confirmatory regression analyses were conducted within the High-ELS sample to examine factors associated with RT-IIV in this subgroup alone; one-tailed p-values were assessed as these analyses tested a specific, directional hypothesis informed by prior findings (Jackson et al., 2012).

To assess the functional validity of our RT-IIV measure, we examined the relation between RT-IIV and subjective cognitive ratings using linear regression. Here, RT-IIV was entered as the dependent variable and MOS-HIV cognitive subscale scores were entered as the independent variable. We also conducted explanatory analyses to examine potential etiological factors associated with brain volume abnormalities, where the potential predictors included neuropsychiatric symptom levels, ELS status, and nadir CD4 levels (a measure of historical HIV-disease severity). Considering our sample size, we focused these analyses on nadir CD4 levels, to the exclusion of other HIV-disease variables (e.g., current CD4, HIVL), given prior data identifying nadir CD4 as one of the primary factors associated with brain volume abnormalities in HIV+ adults (Clark and Cohen, 2010; Cohen et al., 2010; Jernigan et al., 2011; Clark et al., 2012, 2015; Guha et al., 2016). In each of these hierarchical regression models, the brain volume of interest was entered as the dependent variable; scanner type was entered as an initial covariate; neuropsychiatric composite scores were entered as the independent variable in the second step, followed by ELS status in the third step, and nadir CD4 levels in the fourth step. All statistical analyses were conducted using SPSS (version 23, IBM Corporation).

## Results

### Demographic Measures

Demographic data for each group are reported in **Table 1**, including group means and statistics. High-ELS and Low-ELS groups were well matched on several demographic variables including age, estimated premorbid intelligence, lifetime substance use, and HIV-disease factors (*p*s > 0.050) (**Table 1**).

### Neuropsychiatric Measures

The High-ELS group reported significantly higher levels of depression, current stress, and PTSD-related symptoms compared to the Low-ELS group (*t*s ≥ 2.30, *p*s ≤ 0.026) (**Table 1**). Scores on the neuropsychiatric composite index were significantly greater in the High-ELS group than in the Low-ELS group (*t*[41.54] = 3.02, *p* = 0.004) (**Table 1**). This effect was maintained when covarying for age and gender (*p* = 0.002, $\eta_{p}^{2}$= 0.21).

### RT Measures

**FIGURE 2:**
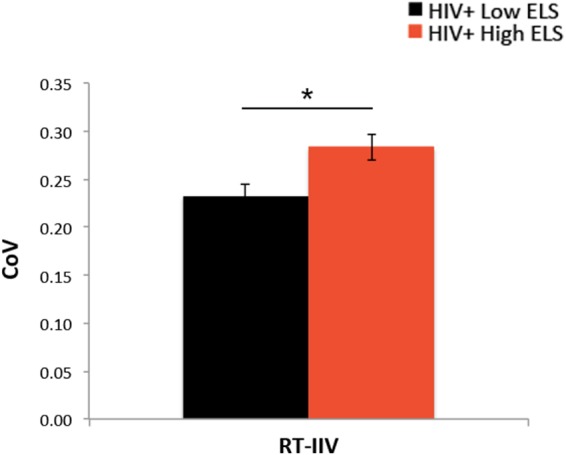
The High-ELS group exhibited greater RT-IIV than the Low-ELS group. ELS, early life stress; RT-IIV, reaction time intra-individual variability; CoV, coefficient of variation, a measure of variability where higher values indicate greater variability. Asterisks indicate that the groups’ means are significantly different at the ^∗^*p* < 0.05 level.

Both groups responded to >96% of trials during the 1-back blocks (**Table 1**). Groups did not differ significantly in 1-back accuracy (A′, **Table 1**). RT latencies were slower on average in the High-ELS than in the Low-ELS group, but this difference was non-significant (**Table 1**). By contrast, the High-ELS group demonstrated significantly greater RT-IIV than the Low-ELS group (*F*[1,39] = 6.80, *p* = 0.013, $\eta_{p}^{2}$= 0.15) (**Figure 2**), even when controlling for neuropsychiatric symptom levels (depression, current stress, PTSD) (*p* = 0.021, $\eta_{p}^{2}$= 0.13). Notably, mean RT-IIV scores in the High-ELS group were one standard deviation higher than in the Low-ELS group. This effect was maintained when covarying for age and gender (*p* = 0.028, $\eta_{p}^{2}$= 0.12).

### Brain Volumes

**FIGURE 3:**
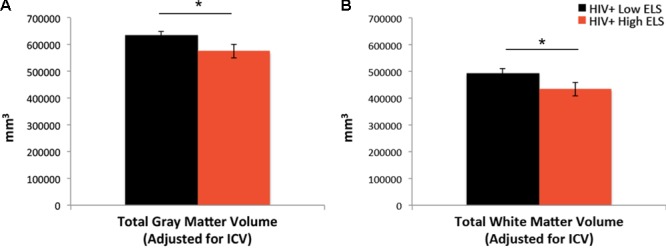
Early life stress (ELS)-related reductions in gray matter **(A)** and white matter **(B)** volumes. ELS, early life stress; ICV, total intracranial volume. Asterisks indicate that the groups’ means are significantly different at the ^∗^*p* < 0.05 level.

Results from the ANCOVA, controlling for neuropsychiatric composite scores and scanner type, revealed that the High-ELS group demonstrated significantly smaller gray and white matter volumes relative to the Low-ELS group (*F*[1,39] = 5.21, *p* = 0.028, $\eta_{p}^{2}$= 0.12; *F*[1,39] = 4.62, p = 0.038, $\eta_{p}^{2}$= 0.11, respectively) (**Figures 3A,B**). Age did not contribute significantly to the models (*p* = 0.408; *p* = 0.943, respectively) and was therefore not included as a covariate in the final analyses. The observed effects were maintained when including gender as a covariate (*p* = 0.047, $\eta_{p}^{2}$= 0.10; *p* = 0.062, $\eta_{p}^{2}$= 0.09, respectively).

### Relation of RT-IIV to Neuropsychiatric Symptoms and Brain Volume Measures

**FIGURE 4:**
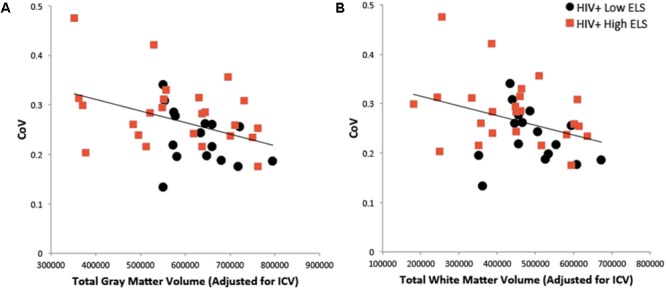
Greater RT-IIV is associated with reduced gray matter **(A)** and white matter **(B)** volumes. ELS, early life stress; RT-IIV, reaction time intra-individual variability; CoV, coefficient of variation; ICV, total intracranial volume.

We examined the relation of RT-IIV to neuropsychiatric symptoms and gray matter volumes using hierarchical regression (**Table 2**). Results revealed a non-significant association with neuropsychiatric symptoms, whereas gray matter volumes were significantly associated with RT-IIV across the entire sample (β = -0.46; *p* = 0.007) (**Figure 4A**). These effects were maintained when including gender as a covariate (β = -0.50; *p* = 0.008). In this analysis, neuropsychiatric symptoms accounted for 1% of the variance in RT-IIV and gray matter volumes accounted for an additional 18%. These findings were replicated when the model was restricted to the High-ELS sample; results from this confirmatory analysis revealed a non-significant association between RT-IIV and neuropsychiatric symptoms (one-tailed *p* = 0.258), whereas associations with gray matter volumes were significant (β = -0.44 [*t* = -1.92, one-tailed *p* = 0.035]). In the High-ELS sample, neuropsychiatric symptoms accounted for <1% of the variance in RT-IIV, and gray matter volumes accounted for an additional 16%.

**Table 2:** Results from the hierarchical regression analysis assessing the relation of RT-IIV to neuropsychiatric symptoms and gray matter volumes (*N* = 40).

Task	*R*^2^	*F*	*p*	Δ*R*^2^	Predictor	β	*t*	*p*
Step 1	0.014	0.54	0.468	0.014	Neuropsychiatric symptoms	0.12	0.73	0.468
Step 2	0.015	0.29	0.749	0.002	Neuropsychiatric symptoms	0.13	0.76	0.451
					Scanner type	0.04	0.24	0.811
Step 3	0.198	2.96	0.045	0.183	Neuropsychiatric symptoms	0.16	1.02	0.317
					Scanner type	–0.11	–0.65	0.521
					Total gray matter volumes	–0.46	–2.86	0.007
Step 4	0.207	2.28	0.080	0.009	Neuropsychiatric symptoms	0.17	1.09	0.283
					Scanner type	–0.11	–0.65	0.523
					Total gray matter volumes	–0.50	–2.83	0.008
					Gender	0.10	0.62	0.542

A similar pattern was observed in the model examining associations between RT-IIV, neuropsychiatric symptoms, and white matter volumes (**Table 3**). Across the entire sample, we observed a non-significant association between RT-IIV and neuropsychiatric composite scores, whereas associations with white matter volumes were significant (β = -0.37; *p* = 0.026) (**Figure 4B**). These effects were maintained when including gender as a covariate (β = -0.42; *p* = 0.028). In this analysis, neuropsychiatric symptoms accounted for 1% of the variance in RT-IIV and white matter volumes accounted for an additional 13%. Results from a confirmatory analysis conducted in the High-ELS group alone revealed a non-significant association between RT-IIV and neuropsychiatric symptoms (one-tailed *p* = 0.284), whereas associations with white matter volumes were significant (β = 0.41 [*t* = -1.79, one-tailed *p* = 0.044]). In the High-ELS sample, neuropsychiatric symptoms accounted for <1% of the variance in RT-IIV, and white matter volumes accounted for an additional 14%.

**Table 3:** Results from the hierarchical regression analysis assessing the relation of RT-IIV to neuropsychiatric symptoms and white matter volumes (*N* = 40).

Task	*R*^2^	*F*	*p*	Δ*R*^2^	Predictor	β	*t*	*p*
Step 1	0.014	0.54	0.468	0.014	Neuropsychiatric symptoms	0.12	0.73	0.468
Step 2	0.015	0.29	0.749	0.002	Neuropsychiatric symptoms	0.13	0.76	0.451
					Scanner type	0.04	0.24	0.811
Step 3	0.144	2.02	0.128	0.129	Neuropsychiatric symptoms	0.15	0.91	0.371
					Scanner type	–0.04	–0.27	0.790
					Total white matter volumes	–0.37	–2.33	0.026
Step 4	0.152	1.57	0.203	0.008	Neuropsychiatric symptoms	0.16	0.98	0.335
					Scanner type	–0.04	–0.25	0.805
					Total white matter volumes	–0.42	–2.29	0.028
					Gender	0.11	0.58	0.564

Associations of RT-IIV to gray and white matter volumes in the Low-ELS group (*r*[13] = -0.349, *p* = 0.102; *r*[13] = -0.166, *p* = 0.278, respectively; one-tailed *p*-values) did not differ significantly from those in the High-ELS group (*r*[21] = -0.378, *p* = 0.038; *r*[21] = -0.358, *p* = 0.047, respectively; one-tailed *p*-values), as indicated by Fisher’s *r*-to-*z* transformations (*z* = 0.09, *p* = 0.928; *z* = 0.59, *p* = 0.555, respectively).

### Relation between RT-IIV and Self-Reported Cognitive Function

**FIGURE 5:**
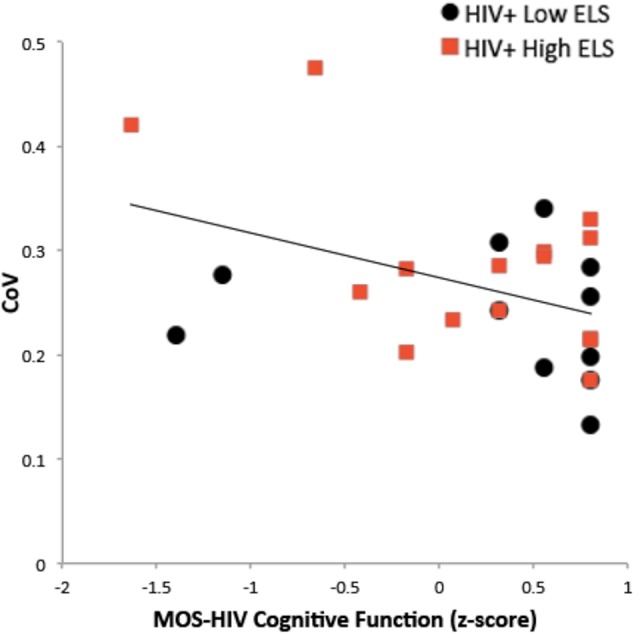
Greater RT-IIV is associated with poorer self-reported cognitive function. RT-IIV, reaction time intra-individual variability; ELS, early life stress; CoV, coefficient of variation; MOS-HIV, The Medical Outcomes Study HIV Health Survey.

Self-report ratings of cognitive function (MOS-HIV) were lower on average in the High-ELS than in the Low-ELS group, but this difference was non-significant (**Table 1**). There was a significant negative association between RT-IIV and subjective ratings of cognitive function (β = -0.42 [*t* = -2.24, *p* = 0.035]) (**Figure 5**), which remained (*p* = 0.035), even when controlling for neuropsychiatric symptoms.

### Predictors of Brain Volume Abnormalities

Results from the exploratory analysis assessing potential etiological predictors of gray matter volume abnormalities (**Table 4**) revealed a non-significant association with neuropsychiatric symptoms (*p* = 0.598), whereas ELS status was a significant predictor of gray matter volumes (*p* = 0.046). When nadir CD4 was added to the model, ELS status (*p* = 0.093) and nadir CD4 (*p* = 0.072) exhibited trend-level effects. In this analysis, neuropsychiatric symptoms accounted for <1% of the variance in gray matter volumes, ELS status accounted for 9%, and nadir CD4 levels accounted for an additional 7%.

**Table 4:** Results from the hierarchical regression analyses assessing the relation of total gray and white matter volumes to neuropsychiatric symptoms, ELS status, and nadir CD4 levels (*N* = 42).

Task	*R*^2^	*F*	*p*	Δ*R*^2^	Predictor	β	*t*	*p*
**Total gray matter volumes**					
Step 1	0.134	6.19	0.017	0.134	Scanner type	–0.37	–2.49	0.017
Step 2	0.140	3.18	0.053	0.006	Scanner type	–0.35	–2.89	0.028
					Neuropsychiatric symptoms	0.08	0.53	0.598
Step 3	0.227	3.72	0.019	0.087	Scanner type	–0.33	–2.70	0.029
					Neuropsychiatric symptoms	0.20	1.27	0.211
					ELS status	–0.32	–2.06	0.046
Step 4	0.292	3.82	0.011	0.065	Scanner type	–0.40	–2.68	0.011
					Neuropsychiatric symptoms	0.14	0.86	0.396
					ELS status	–0.26	–1.73	0.093
					Nadir CD4 cell count	0.27	1.85	0.072
**Total white matter volumes**					
Step 1	0.070	2.99	0.091	0.070	Scanner type	–0.26	–1.73	0.091
Step 2	0.075	1.58	0.220	0.005	Scanner type	–0.25	–2.57	0.125
					Neuropsychiatric symptoms	0.07	0.47	0.643
Step 3	0.158	2.38	0.085	0.083	Scanner type	–0.23	–1.52	0.137
					Neuropsychiatric symptoms	0.19	1.16	0.253
					ELS status	–0.31	–1.94	0.060
Step 4	0.193	2.21	0.087	0.035	Scanner type	–0.27	–1.76	0.086
					Neuropsychiatric symptoms	0.14	0.85	0.398
					ELS status	–0.27	–1.67	0.104
					Nadir CD4 cell count	0.20	1.26	0.215

We observed a similar pattern in the model predicting total white matter volumes (**Table 4**), where associations with neuropsychiatric symptoms were non-significant (*p* = 0.643), while trend-level associations were observed with ELS status (*p* = 0.060). When nadir CD4 was added to the model, a non-significant association between nadir CD4 and white matter volumes was observed (*p* = 0.215), and the association between ELS status and white matter volumes rose just above the trend level (*p* = 0.104). In this analysis, neuropsychiatric symptoms accounted for <1% of the variance in white matter volumes, ELS status accounted for 8%, and nadir CD4 levels accounted for an additional 4%.

## Discussion

The current study examined the hypothesis that cognitive difficulties observed in HIV+ High-ELS adults reflect brain volume abnormalities rather than neuropsychiatric symptoms. Several key findings emerged from this study. First, we observed that High-ELS adults exhibited greater cognitive difficulties than those with low ELS, as indexed by RT-IIV. This finding supports prior data indicating that high ELS exposure is associated with cognitive dysfunction in HIV+ adults (Clark et al., 2012; Spies et al., 2012). Considering that high ELS exposure in HIV+ adults is also associated with elevated neuropsychiatric symptoms (Myers et al., 2006; Clark et al., 2017), which are known to be associated with elevations in RT-IIV (Ode et al., 2011; Swick et al., 2012), we examined whether RT-IIV elevations in the High-ELS group were driven by neuropsychiatric symptoms (depression, current stress, PTSD). While the High-ELS group did indeed demonstrate elevated neuropsychiatric symptom levels, group differences in RT-IIV persisted even after controlling for neuropsychiatric symptom levels. These data suggest that ELS-related elevations in RT-IIV are not driven by increased neuropsychiatric symptoms.

By contrast, results from our neuroimaging analyses suggest that ELS-related elevations in RT-IIV are strongly linked to global brain volumes. As expected, RT-IIV elevations were significantly associated with total gray and white matter volume reductions across the entire sample. When examining the High-ELS group alone, we found that measures of total gray and white matter volumes accounted for more than 13% of the variance in RT-IIV, whereas neuropsychiatric symptoms accounted for less than 1%. Accordingly, these results demonstrate that, although the High-ELS sample experiences elevated neuropsychiatric symptoms, these symptoms contribute minimally to RT-IIV elevations. These findings thus support our hypothesis that, in HIV+ High-ELS adults, ELS-related RT-IIV elevations are more strongly associated with global brain volume reductions than with neuropsychiatric symptom elevations. Such results extend prior data indicating strong associations between ELS-related cognitive difficulties and regional brain volume abnormalities in HIV+ samples (Clark et al., 2012; Spies et al., 2012).

Our study also revealed the novel finding that HIV+ High-ELS adults demonstrated lower total gray and white matter volumes than HIV+ Low-ELS adults, consistent with prior reports of regional gray matter abnormalities in HIV+ High-ELS adults (Clark et al., 2012; Spies et al., 2016). Notably, ELS-related white matter volume abnormalities and reductions in global gray matter volumes in HIV+ samples have not been reported previously. Our data thus suggest that high ELS exposure may have a broader impact on neural structure in HIV+ adults than was previously understood. Indeed, when we examined potential mechanistic factors associated with these effects, we found that volumetric reductions were more strongly associated with ELS status than with neuropsychiatric symptoms. Moreover, ELS status and nadir CD4 levels explained similar amounts of variance in brain matter volumes. Many studies have identified nadir CD4 levels as a critically important historical factor impacting neural outcomes in HIV+ adults (Clark and Cohen, 2010; Cohen et al., 2010; Jernigan et al., 2011; Clark et al., 2012, 2015; Guha et al., 2016). Our current findings suggest that high ELS exposure may be as important to consider as nadir CD4 levels when examining factors that impact neural outcomes in HIV+ adults.

As noted above, we found that RT-IIV exhibited significant associations with global measures of both gray and white matter volumes across the entire sample. The strength of these associations did not differ significantly between High-ELS and Low-ELS groups in the current sample; however, additional studies with larger sample sizes are needed to provide greater certainty regarding this finding. To our knowledge, this is the first study to demonstrate that RT-IIV is sensitive to structural brain abnormalities in HIV+ individuals. Such findings build on prior results indicating that RT-IIV is sensitive to global reductions in white matter volume and integrity in non-HIV samples (Moy et al., 2011; Jackson et al., 2012). It is more novel, however, to find significant correlations between RT-IIV and gray matter volumes. For example, one recent study conducted in a non-demented, non-HIV cohort did not observe significant associations between RT-IIV and gray matter density (Moy et al., 2011). It is likely that this inconsistency reflects differences in the samples under investigation, where disease-related processes that alter brain-behavior relations may be driving the current findings. Indeed, prior studies have observed significant correlations between regional MRI volumes and RT-IIV in adults with mild cognitive impairment (MCI) that were not present in cognitively intact individuals (Anstey et al., 2007). Such findings suggest that stronger brain-behavior associations may arise within the context of a neuropathological disease process, such as MCI, or in the case of the current study, HIV and/or high ELS exposure.

Although RT-IIV is considered to be a strong indicator of neural dysfunction, the exact neural underpinnings of increased RT-IIV are unclear. For example, RT-IIV has been linked to frontal lobe activation (Bellgrove et al., 2004), activation of the left anterior cingulate (Johnson et al., 2015), frontal-lobe circuitry (Chuah et al., 2006), frontal-lobe white matter hyperintensity burden (Bunce et al., 2007), widespread white matter integrity (Fjell et al., 2011), dopamine-mediated neurotransmission (MacDonald et al., 2006; Grant et al., 2014), and default mode network suppression (Weissman et al., 2006; Kelly et al., 2008). Such findings suggest that the neural etiology of elevated RT-IIV may be multifactorial. In this context, our observation of significant associations between elevated RT-IIV and global gray and white matter volumes is consistent with the proposition that RT-IIV is a marker of overall neurological integrity. It has not yet been determined whether ELS exposure potentiates HIV-related neural abnormalities or whether it is associated with independent pathophysiological mechanisms (Womersley et al., 2017). Hence, our findings provide foundational evidence for future investigations that seek to examine specific ELS-related and HIV-related neural mechanisms underlying RT-IIV elevations in HIV+ High-ELS adults. With prior reports of ELS-related abnormalities in default mode network suppression (Philip et al., 2013), and HIV-related frontostriatal (Melrose et al., 2008; Ipser et al., 2015), dopaminergic (Berger et al., 1994; Kumar et al., 2009), and white matter abnormalities (Pomara et al., 2001; Tate et al., 2011; Robinson-Papp et al., 2017), several neural mechanisms could be implicated.

Some limitations of this study should be noted, with implications for future research. Although our sample size was on par with prior behavioral studies of RT-IIV in HIV+ samples (Levine et al., 2006; Ettenhofer et al., 2010), the size of our groups was somewhat small for a volumetric MRI study. Nevertheless, we were able to detect ELS-related differences in gray and white matter volumes, as well as associations between RT-IIV and brain volume reductions in the High-ELS sample. Replication of our findings in larger samples, which offer greater statistical power and the ability to further examine the effects of potential moderating factors (e.g., age, gender), would provide additional certainty regarding the reported observations. Similarly, studies that compare HIV+ to HIV- adults are needed to elucidate potential independent and combined effects of HIV infection and high ELS on the observed outcomes. Second, this study did not include a full neuropsychological battery, and it was thus not possible to test whether our RT-IIV measure strongly reflects cognitive function, as indicated by objective measures. Prior data, which point to a strong association between global cognitive impairment and RT-IIV in HIV+ adults (Ettenhofer et al., 2010), support our use of RT-IIV as a cognitive measure. Moreover, our observation of a significant association between RT-IIV and subjective ratings of cognitive function provides further evidence of its validity as a cognitive marker.

## Conclusion

We report that HIV+ High-ELS adults demonstrate greater cognitive difficulties (RT-IIV), greater neuropsychiatric symptoms, and reduced global brain volumes relative to those with Low-ELS. Moreover, we report that, in HIV+ High-ELS adults, ELS-related cognitive difficulties (RT-IIV) exhibit strong associations with global brain volumes, whereas ELS-related elevations in neuropsychiatric symptoms appear to contribute minimally to these cognitive difficulties. Such findings add to a rapidly expanding literature indicating that early environmental experiences can have long-term effects on the structure and function of the human brain (McEwen, 2008; Clark et al., 2012, 2017; Philip et al., 2013; Spies et al., 2016; Teicher and Samson, 2016; Thames et al., 2017). Future studies should thus be conducted to better understand how ELS-related pathophysiological mechanisms contribute to the development of volumetric and other neural abnormalities in HIV+ adults. Such studies have the potential to provide greater insights into possible targets for therapeutic intervention.

## Ethics Statement

This study was carried out in accordance with the recommendations of the Institutional Review Boards at the Icahn School of Medicine at Mount Sinai and The Miriam Hospital. All subjects gave written informed consent in accordance with the Declaration of Helsinki. The protocol was approved by the Institutional Review Boards at the Icahn School of Medicine at Mount Sinai and The Miriam Hospital.

## Author Contributions

UC, MAR, and SM: substantial contributions to the conception or design of the work; UC and RH: acquisition, analysis, or interpretation of data for the work; UC, MAR, RH, and SM: drafting the work or revising it critically for important intellectual content, final approval of the version to be published, agreement to be accountable for all aspects of the work in ensuring that questions related to the accuracy or integrity of any part of the work are appropriately investigated and resolved.

## Conflict of Interest Statement

The authors declare that the research was conducted in the absence of any commercial or financial relationships that could be construed as a potential conflict of interest. The reviewer LC and handling Editor declared their shared affiliation.

## Footnotes

1 www.pstnet.com
