# Supplementary material for: Early Life Stress-Related Elevations in Reaction Time Variability Are Associated with Brain Volume Reductions in HIV+ Adults
Source: Front Behav Neurosci. 2018 Jan 30;12:6. doi: 10.3389/fnbeh.2018.00006 (PMC5797588; doi:10.3389/fnbeh.2018.00006)

**Supplemental Figure S1**

Across the sample, scores on the first component identified through principal components analysis are plotted against neuropsychiatric composite z-scores, demonstrating the correlation between the two.


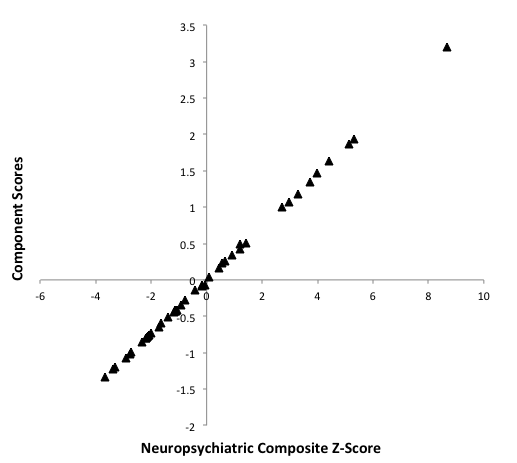

Supplement: Supplementary file 1 [file Data_Sheet_1.docx]
